# Supplementary material for: Predicting visual function by interpreting a neuronal wiring diagram
Source: Nature. 2024 Oct 2;634(8032):113–23. doi: 10.1038/s41586-024-07953-5 (PMC11446822; doi:10.1038/s41586-024-07953-5)
Supplement: Supplementary file 6 — CRF and ERF predictions for individual TmY4 and TmY9 cells. Analogous to Supplementary Data 3, but for TmY target types. Shown are the top four monosynaptic pathways, the strongest pathway passing through each of the top ten intermediary types (ranking from Extended Data Fig. 7), and the trisynaptic pathway Tm1–TmY–Dm3–TmY (see the section entitled Prediction of spatial normalization). [file 41586_2024_7953_MOESM6_ESM.zip › DataS4/TmY9qΓèÑ/720575940630585820.pdf]

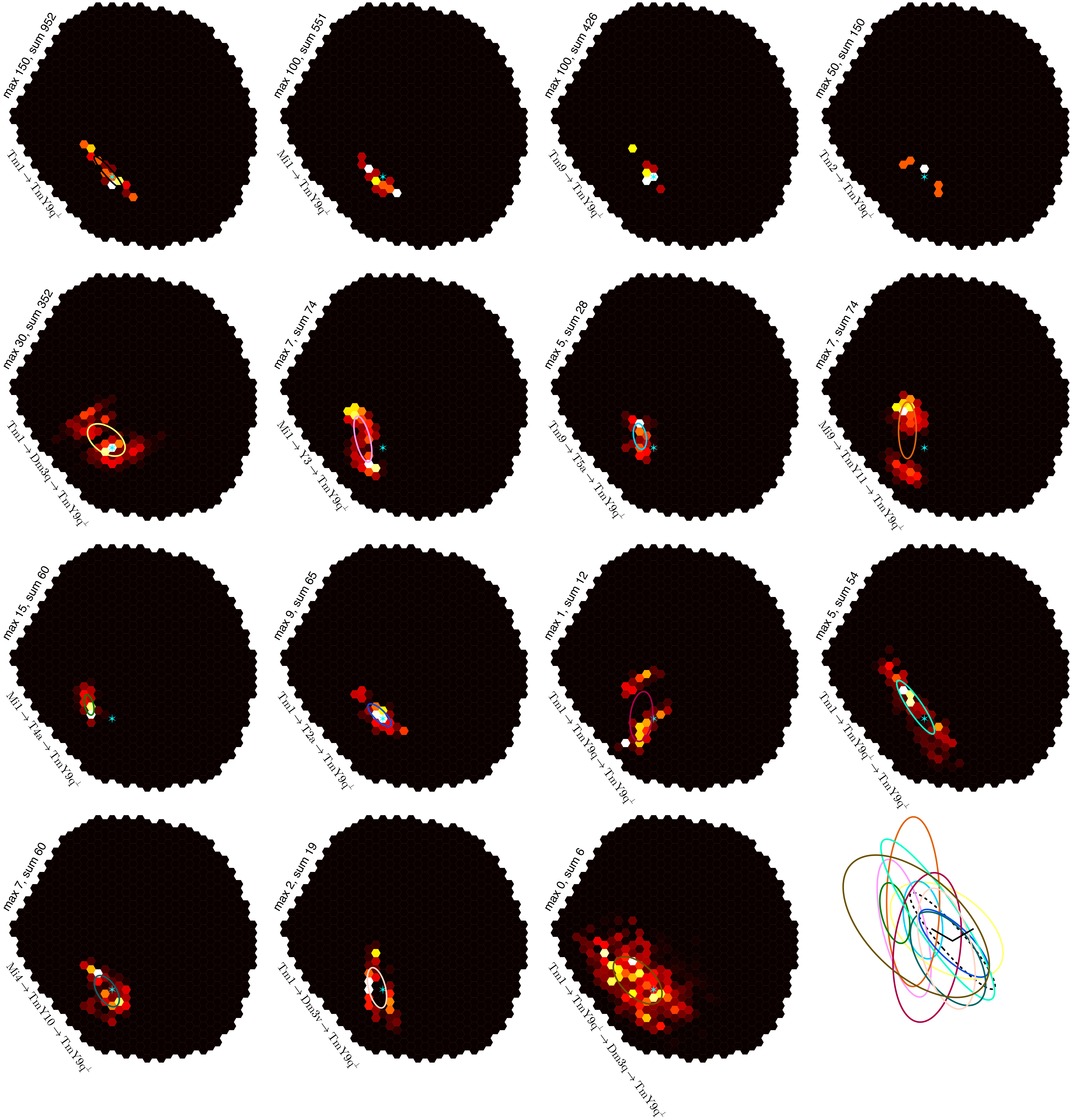

max 150, sum 952
Tm1 → TmY9q⊥
max 100, sum 551
Mi1 → TmY9q⊥
max 100, sum 426
Tm9 → TmY9q⊥
max 50, sum 150
Tm2 → TmY9q⊥
max 30, sum 352
Tm1 → Dm3q → TmY9q⊥
max 7, sum 74
Mi1 → Y3 → TmY9q⊥
max 5, sum 28
Tm9 → T5a → TmY9q⊥
max 7, sum 74
Mi9 → TmY11 → TmY9q⊥
max 15, sum 60
Mi1 → T4a → TmY9q⊥
max 9, sum 65
Tm1 → T2a → TmY9q⊥
max 1, sum 12
Tm1 → TmY9q → TmY9q⊥
max 5, sum 54
Tm1 → TmY9q⊥ → TmY9q⊥
max 7, sum 60
Mi4 → TmY10 → TmY9q⊥
max 2, sum 19
Tm1 → Dm3v → TmY9q⊥
max 0, sum 6
Tm1 → TmY9q⊥ → Dm3q → TmY9q⊥
